# A hidden site: positron emission tomography-computed tomography (PET-CT) unveiling subclinical masseter involvement in follicular lymphoma – case report

**DOI:** 10.11604/pamj.2025.52.96.49728

**Published:** 2025-11-04

**Authors:** Sana Ferchichi, Hamida Khémiri Guerbouj, Zahra Jouini, Ghada Kharrat, Wafa Skouri, Haifa Tounsi, Raja Amri

**Affiliations:** 1 Department of Otorhinolaryngology, Ear and Head and Neck (ENT) Surgery, Taher Maâmouri Hospital, Nabeul, Tunisia,; 2 Faculty of Medicine of Tunis, University of Tunis El Manar, Tunis, Tunisia,; 3 Department of Internal Medicine, Taher Maâmouri Hospital, Nabeul, Tunisia

**Keywords:** Lymphoma, positron emission tomography, extranodal disease, muscle involvement, case report

## Abstract

Extranodal lymphoma involvement refers to lymphoma arising outside primary lymphatic organs. Muscular involvement is rare. This case report highlights the critical role of positron emission tomography-computed tomography (PET-CT) in detecting underestimated extranodal lesions, especially in muscles. We report the case of a 66-year-old patient with no prior significant medical history who presented with 3 months of cervical lymphadenopathy, asthenia, and night sweats. Examination revealed supracentimetric elastic cervical and inguinal lymphadenopathy and splenomegaly. Computed tomography suggested nodal disease above and below the diaphragm. Biopsy confirmed grade 3B follicular lymphoma and bone marrow infiltration. Positron emission tomography-computed tomography revealed intense fluorodeoxyglucose (FDG) uptake in nodal regions, spleen, bone marrow, and left masseter muscle, which was not visible on CT. The disease was staged as Ann Arbor stage IV, and the patient was planned for 8 cycles of R-CHOP and intrathecal methotrexate. As a final point, ^18^F-FDG PET/CT is indispensable for accurate staging and assessment of treatment response in follicular lymphoma, permitting detection of extranodal disease not visible on CT.

## Introduction

Lymphomas are a heterogeneous group of over 50 subtypes, broadly divided into Hodgkin and non-Hodgkin lymphoma (NHL) [1]. Follicular lymphoma is a B-cell NHL. Extranodal involvement occurs in 25-40% of lymphomas [1], with muscular involvement being especially rare (~1.4%) [2]. We present a rare case of disseminated G3B FL with subclinical masseter involvement detected only by PET-CT.

## Patient and observation

**Patient information:** a 66-year-old patient with no noteworthy past medical history presented with cervical lymphadenopathy, asthenia, night sweats, and 4kg weight loss over 3 months. There was no fever.

**Clinical findings:** on examination, supracentimetric, elastic cervical and inguinal lymphadenopathy and splenomegaly were found; other systems were unremarkable.

**Timeline of the current episode:** the patient first noticed neck swelling and fatigue approximately three months before seeking medical attention. In the following weeks, symptoms worsened, including night sweats and unintentional weight loss. He sought medical attention when the lymphadenopathy became more prominent and persistent. Following an initial consultation with a general practitioner, the patient was referred to our department for specialized care. Initial clinical evaluation and imaging studies led to further diagnostic workup, which confirmed the diagnosis and initiated treatment shortly thereafter.

**Diagnostic assessment:** laboratory tests showed leukocytosis (white blood cells (WBC) 16,810/mm^3^, lymphocytes 12,110/mm^3^), normocytic regenerative anemia (Hb 7.3g/dL), and elevated lactate dehydrogenase (LDH). Serologies for HIV, Hepatitis B, and C were negative. Computed tomography scan demonstrated lymphadenopathy above and below the diaphragm, and splenomegaly.

**Figure 1:**
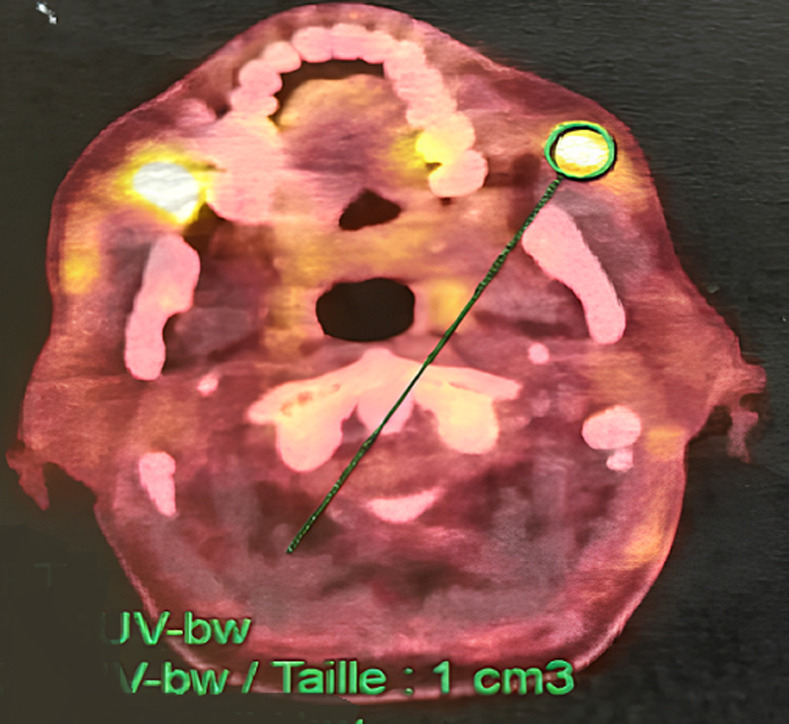
axial positron emission tomography-computed tomography section showing intense fluorodeoxyglucose uptake in the left masseter muscle, consistent with subclinical lymphomatous involvement

**Diagnosis:** excisional lymph node biopsy confirmed Grade 3B follicular lymphoma. Bone marrow biopsy showed marrow infiltration. Positron emission tomography-computed tomography revealed intense FDG uptake in nodal regions, spleen, bone marrow, and left masseter muscle (Figure 1). On CT, the masseter was isodense before and after contrast, making the lesion invisible alone. The disease was classified as Ann Arbor Stage IV, FLIPI score of 4, and IPI score of 2.

**Therapeutic interventions:** following a multidisciplinary team discussion, the patient was scheduled to receive systemic chemotherapy combined with intrathecal methotrexate.

**Follow-up and outcomes:** the patient underwent 8 cycles of R-CHOP chemotherapy and received 4 intrathecal methotrexate injections. The treatment was well tolerated overall, with minor side effects including fatigue and mild nausea, and no major complications were reported. Post-treatment imaging confirmed complete metabolic remission, and at 24-month follow-up, the patient remained clinically stable and disease-free.

**Patient perspective:** the patient expressed relief and gratitude upon receiving news of complete remission. He reported feeling well throughout the treatment period, despite minor side effects, and appreciated the support of the medical team. At his follow-up visit, he remained optimistic and satisfied with the care he received.

**Informed consent:** the patients gave fully informed and written consent for publication.

## Discussion

Lymphoma is a neoplastic proliferation of lymphoid cells at various stages of differentiation, primarily affecting the lymph nodes, with potential infiltration into key lymphatic organs, including the bone marrow, spleen, and thymus. Extranodal involvement, occurring in approximately 25-40% of cases, is defined as lymphoma arising outside these primary lymphatic structures [1]. In clinical practice, distinguishing between primary extranodal lymphoma and disseminated nodal lymphoma with secondary extranodal involvement remains debatable. Broadly, primary extranodal lymphoma refers to cases where the extranodal site is the principal location of the disease, as well as those involving an extranodal organ along with its associated draining lymph nodes [3]. Muscular lymphoma is rare; it accounts for 1.4% [2]. Lymphomatous muscle involvement occurs through three distinct mechanisms: direct extension from adjacent affected lymph nodes, secondary metastatic spread, and primary muscle lymphoma, which remains exceedingly rare [4]. In our patient, it is likely a metastatic dissemination. In this case, lymphoma typically progresses by infiltrating malignant cells that lack extracellular stroma and intercellular junctions, leading to diffuse enlargement of affected structures and the ability to cross fascial boundaries [5]. The most commonly affected muscles are those of the extremities, pelvis, and gluteal regions. However, the involvement of the masseter muscle, as observed in our case, is atypical [6]. The involvement of the masseter muscle may clinically present with localized soft tissue swelling or pain, difficulty chewing, trismus, and/or trigeminal neuralgia. In our case, however, masseter involvement was subclinical, which is extremely rare. In imaging, muscular lymphoma does not have distinct sonographic features; however, soft tissue lesions generally appear solid, hypoechoic, and may have well or poorly defined borders [1]. Muscle enlargement, either as a discrete mass or diffuse muscle infiltration, can be seen on CT and magnetic resonance imaging (MRI). The CT density may be similar to or slightly decreased compared to unaffected muscles, with minimal enhancement after intravenous contrast injection [5].

Likewise, on MRI, abnormal tissue may appear isointense on T1-weighted images compared to the surrounding muscle. Contrast enhancement may reveal peripheral thick band-like enhancement or marginal septal enhancement [4]. Positron emission tomography-computed tomography has established itself as the preferred imaging modality for staging, restaging, and monitoring treatment response in lymphoma patients. The presence of focal areas with intense FDG uptake, whether singular or multiple, with or without associated CT changes, should raise suspicion for lymphomatous muscle involvement [4]. When CT imaging does not show abnormalities, PET-CT can assist clinicians in selecting appropriate biopsy sites, especially when muscle lesions represent the sole extranodal site of involvement. The SUVmax of muscle lesions in lymphoma can vary considerably, primarily influenced by the histopathologic grade of the lymphoma [4]. When staging lymphoma, PET-CT allows for the detection of extranodal sites that may appear normal on contrast-enhanced computed tomography (CECT) [7]. It also differentiates lymphomatous infiltration from benign causes of increased FDG uptake, ensuring more accurate disease staging. In the study by Othman *et al*. PET-CT led to upstaging in 10% and downstaging in 5% of cases. In a retrospective study involving 45 patients with untreated, biopsy-proven follicular lymphoma, PET-CT identified significantly more nodal (+51%) and extranodal (+89%) lesions compared to CT [7]. Additionally, PET-CT modified the Ann Arbor staging in eight patients (18%) [8]. The PET-CT is a highly sensitive imaging technique for detecting hypermetabolic areas in muscles and has a considerable degree of specificity. It can effectively distinguish FDG uptake caused by benign factors (recent muscle activity, inflammation, infection), which is typically symmetric, diffuse, and reversible, with a moderate to low SUVmax (usually < 3-5) [7]. Indeed, in the research of Albano *et al*. it has been demonstrated that G3BFL has specific PET-CT features in terms of average standardized uptake value (SUV), metabolic tumor volume (MTV), and total lesion glycolysis (TLG) compared to low-grade FL and DLBCL [9]. It has been demonstrated that, in addition to visual analysis, semi-quantitative PET biomarkers such as SUV, MTV, and TLG help define different disease characteristics, such as the intensity of uptake, tumor burden, and disease dissemination [9]. Similarly, Barraclough *et al*. have demonstrated that the complete metabolic response at the end of treatment of G3BFL holds prognostic significance and is associated with improved progression-free survival and overall survival [10].

## Conclusion

In this rare case of G3B FL, PET-CT revealed subclinical masseter muscle involvement not visible on CT. This underscores the essential role of ^18^F-FDG PET/CT in accurate staging and guiding management in follicular lymphoma.
